# Effect of exposure to different light colors on embryonic development and neurophysiological traits in the chick embryo

**DOI:** 10.14202/vetworld.2021.1284-1289

**Published:** 2021-05-24

**Authors:** S. M. Abdulateef, M. A. Al-Bayar, A. A. Majid, S. S. Shawkat, A. Tatar, M. Q. Al-Ani

**Affiliations:** 1 Department of Animal Production, College of Agriculture, University of Anbar, Ramadi, Anbar, Iraq; 2 Department of Animal Sciences, College of Agricultural Sciences, University of Sulaimani, Kurdistan, Iraq; 3 Animal Science Research Department, Golestan Agricultural and Natural Resources Research and Education Center, AREEO, Gorgan, Iran; 4 Department of Biology, College of Science, University of Anbar, Ramadi, Anbar, Iraq

**Keywords:** embryo, embryonic development, light color, neurophysiology

## Abstract

**Background and Aim:**

Many environmental factors exist that influence embryonic development which is missing in the poultry industry, such as light in incubation facilities or hatcheries. Light plays an important role in the growth and development of chick embryos, whereas dark environments can lead to hatching failure or embryo distortion. Therefore, this study aimed to demonstrate the importance of light and its various colors on the growth and development of broiler chick embryos.

**Materials and Methods:**

Four treatments were used to study the impact of various light colors on the growth of embryos and their neurophysiological traits: Dark without light (D), red light (RL), blue light (BL), and green light (GL), with three replicates per treatment (25 eggs/replicate) for a total of 300 fertile Ross 308 eggs. Each treatment was assigned to one incubator (75 eggs/incubator), whereas all other conditions were kept the same.

**Results:**

The results showed a significant increase (p<0.01) in embryonic development for embryo weight, chick body weight, hatchability, and embryo index for RL, BL, and especially GL. RL, BL, and especially GL significantly increased (p<0.01) neurophysiological traits of the neurons, brain weight, and brain index.

**Conclusion:**

The use of light during the embryonic period affects the development of the embryo and its neurophysiological traits.

## Introduction

The poultry industry discovered that an increase in light intensity can accelerate embryonic development [1,2]. In nature, chicken embryos at different ages and species certainly receive some light during incubation, whereas in industrial hatcheries, eggs are incubated in the dark for 21 days. Several studies demonstrated that exposing fertile eggs to light can increase the growth of the embryo and decrease the incubation period [2-4]. Post-hatch artificial lighting is an important management method for the growth and muscle development of meat-type birds [5]. Embryo exposure to light leads to changes in metabolic rate [6], such as an increase in embryo metabolic rate in the pigeon *Columba livia* in light compared with darkness [7]. Furthermore, exposure to light increased the heart rate of embryos [8,9].

Providing light during broiler egg incubation affects production, health, and exhibits the potential to reduce stress associated with growth and production [10]. Archer [11] reported that providing light for 12 h/day during egg incubation reduced susceptibility to stress of broilers post-hatch. The physiological mechanism of light stimulating embryonic growth and development differs before and after formation and maturation of the retinal photoreceptor. The hypothalamic pacemaker and pineal gland are the main parts of the circadian avian system [12]. Embryonic cell proliferation increases with high light intensity [13]. Mussttaf [14] reported that visible light affects cellular metabolism regulated by cyclic adenosine monophosphate levels, which results in DNA synthesis. Experimental work in domesticated species of birds showed that light accelerates embryonic development by increasing metabolic activity and increased embryonic development, pineal gland formation, and modifies melatonin synthesis [15].

Low light intensity (e.g. 10 lx) can entrain embryonic starlings *Sturnus valgus* [16] to show a light-dark rhythm mediated by high melatonin hormone concentrations in darkness and low levels during light exposure, which is a universal feature of embryonic organisms[16]. González-Candia [17] demonstrated that chicken embryos exposed to light for as little as 1 h decreases melatonin production and affects embryonic development.

This study aimed to assess the effect of exposing fertile Ross 308 eggs to red, blue, and green light (GL) on embryo weight at 19 days of incubation, percentage of hatchability, neuron diameter, brain weight, percentage of brain weight, and percentage of embryo weight.

## Materials and Methods

### Ethical approval

The study was conducted in accordance with the protocol authorized by the University of Anbar, Ethics Committee, Iraq. Fertile eggs from Ross (308) strain broiler breeder hens were obtained from a commercial farm.

### Study period and location

This study was carried out from 10-10-2019 to 20-11-2019 at the Department of Animal Sciences, College of Agricultural Sciences, University of Sulaimani, Iraq.

### Experimental study

Four egg incubators were used (Cimuka, Turkey). Each incubator was used to incubate eggs using one specific light color according to the following experimental treatments: Dark without light (D) (control), red light (RL), blue light (BL), and GL. Three replicates for each treatment and 75 eggs per treatment (25 egg/replicate) were present for a total of 300 fertile Ross 308 eggs. Apart from the treatment, all conditions were kept equal across incubators including, temperature, CO_2_ concentration, humidity, and ventilation.

### Light control

In this study, a 12:12 light: dark period was provided using electric LEDs (intensity =560 nm, 0.1 watts/m^2^ at eggshell level). The light was provided from 1 to 21 days of incubation.

### Study traits

#### Egg weight, embryo weight, chick weight, and hatchability

The egg weight in the control and experimental groups ranged from 50 to 60 g and was similar across all treatment groups. The embryo was separated according to Abdulateef [18] on 19^th^ day of hatching, and the embryo weight was recorded. After hatching, the chicks from each treatment were collected and weighed. The hatchability was determined as the number of chicks hatched divided by the number of fertile eggs set.

### Embryo index (EI)

All eggs from each treatment group were weighed individually at the end of the experiment. Next, the embryos were separated, cleaned, and weighed. The EI for all eggs was calculated using the formula below [19].


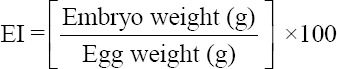


### Neurophysiological traits

#### Brain tissue collection

Chicks were treated with ether (anesthetic or hypnotic liquid), and after anesthesia, they were euthanized. They were dissected, and the brain was separated from the skull by cutting all the cranial nerves and vessels at the base. The weight of the entire brain was measured.

### Tissue processing

Immediately after the tissue was obtained, it was fixed by immersion in 4% paraformaldehyde at 4°C for 2 weeks. The brains were dehydrated, infiltrated, and blocks were prepared by embedding in Paraplast. Serial coronal sections of 7 mm thickness were cut with a rotary microtome. The sections were mounted on egg albumin-coated glass slides and subsequently stained for Nissl substance with 1% buffered thionin. The size of the sections at a distance of 2 mm from the rostral end of the brain was compared between treatment groups [20].

### Quantification

The measurement of the neuronal nuclear area was determined using an image analyzing system (Axio Zeizz, USA). The measurements were made under a 100× objective lens with a pixel size of 0.51 mm.

### Brain index (BI)

At the end of the experiment, the hatching chicks from each treatment group were collected and weighed. As mentioned above, the brain was extracted, and the whole brain was weighed. The BI was calculated according to the following formula:


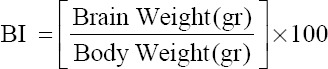


### Statistical analysis

Data were analyzed as a complete randomized design using the SAS program (SAS Institute, USA) for statistical analysis [21]. The mean for each treatment group was compared for significant differences using Duncan’s polynomial [22]. The level of significance was set at p<0.01.

## Results

Table-1 shows the effect of exposure to different light colors on embryonic development and hatchability of chick embryos. A significant increase was found (p<0.01) in embryo weight (19 days incubation) for RL, BL, and GL (33.55, 33.93, and 34.70 g, respectively) compared with D (30.00 g). In addition, a significant increase was found (p<0.01) in body weight (1 old day after hatching) for GL and BL (41.23 and 40.23 g, respectively) compared with RL and D (38.90 and 34.73 g, respectively), whereas RL also resulted in significantly increased body weight (p<0.01) compared with D. However, Table-1 shows that hatchability was significantly higher (p<0.01) in GL (77.00%) compared with the other treatments D, RL, and BL (66.66%, 69.00%, and 72.0%, respectively). BL exhibited significantly higher hatchability compared with D and RL, but no difference was found between RL and D.

**Table-1:** The effect of exposure to different light colors on chick embryonic development and hatchability.

Treatments	Egg weight (g)	Embryo weight* (g)	Chick weight (g)	Hatchability (%)
D	56.71	30.00^b^	34.73^c^	66.66^c^
RL	56.62	33.53^a^	38.90^b^	69.00^c^
BL	56.36	33.93^a^	40.23^a^	72.00^b^
GL	56.21	34.70^a^	41.23^a^	77.00^a^
Mean	56.47	33.04	38.77	71.16
**SEM	0.14	0.56	0.76	1.20
Significant	***NS	0.0001	0.0001	0.0001

* Embryo weight at 19 days,

** SEM=Standard error mean,

*** NS=Non-significant, ^a,b,c^Means in the same columns with different superscripts differ significantly. D=Control (without light), RL=Red light, BL=Blue light, and GL=Green light

Table-2 shows the effect of exposure to different light colors on neurophysiological traits in the chick embryo. Significant increases were found (p<0.01) in neurons ranking from highest to lowest treatment: GL (45.63 μm), BL (43.03 μm), RL (40.40 μm), and D (30.20 μm). An effect of exposure to different light colors on brain weight was also observed (Table-2). No difference was found between BL and GL (0.88 and 0.91 g, respectively), but they were significantly higher (p<0.01) compared with RL and D (0.86 and 0.75 g, respectively). D demonstrated the lowest brain weight (p<0.01).

**Table-2:** The effect of exposure to different light colors on neurophysiological traits in chick embryos.

Treatments	Egg weight (g)	Neurons micron (μm)	Brain weight (g)
D	56.71	30.20^d^	0.75^c^
RL	56.62	40.40^c^	0.86^b^
BL	56.36	43.03^b^	0.88^a^
GL	56.21	45.63^a^	0.91^a^
Mean	56.47	39.81	0.84
*SEM	0.14	1.78	0.01
Significant	**NS	0.0001	0.0001

* SEM=Standard error mean,

** NS=Non-significant, ^a,b,c^Means in the same columns with different superscripts differ significantly. D=Control (without light), RL=Red light, BL=Blue light, and GL=Green lights

**Figure-1:**
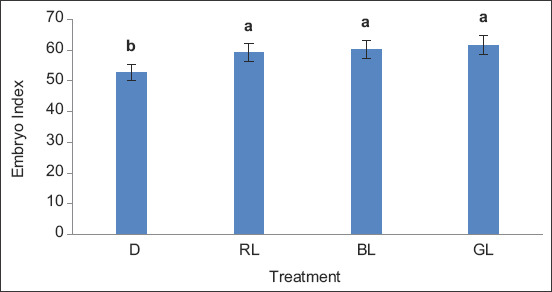
The effect of exposure to different light colors on embryo index. D=Control (without light), RL=Red light, BL=Blue light and GL=Green light.

**Figure-2:**
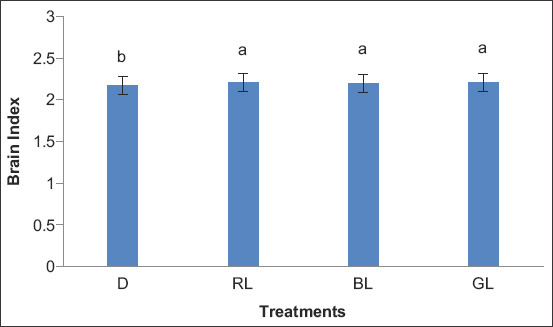
The effect of exposure to different light colors on brain index. D=Control (without light), RL=Red light, BL=Blue light and GL=Green light.

Figure-1 illustrates the effect of exposure to different light colors on the EI. The EI was significantly higher (p<0.01) in RL, BL, and GL (59.24, 60.20, and 61.74, respectively) compared with D (52.90). Exposure to different light colors also affected the BI (Figure-2). BI was significantly higher (p<0.01) in RL, BL, and GL (2.21, 2.20, and 2.21, respectively) compared with D (2.17).

## Discussion

Light plays an important role in the growth and development of living organisms [23-25]. In many animals, light is a basic necessity for embryonic development and is one of the environmental factors that influence embryo growth rate, hatchability, and hatching time, whereas dark conditions may result in hatching failure or distortion of the embryo [6,26,27]. Light stimulation can improve embryonic development by increasing embryonic metabolism and impact chick phenotypes, such as patterns of lateralization in birds [28]. However, light is often overlooked in industrial hatcheries as an ecological factor during the incubation of avian eggs. Therefore, this study was conducted to investigate the importance of light as a key factor in embryonic development during incubation.

Two possible mechanisms exist underlying the effect of light on the embryo. First, light may promote developmental rates by increasing the metabolic rate of embryos. The increase in embryonic heart rate (an important metabolic index) under light conditions indicates that this mechanism may occur [29,30]. Second, evidence exists that visual stimulation before hatching leads to accelerated embryonic development of the embryo [31]. The vision of birds is superior, and the color of light, in addition to the light regimen and intensity, may also impact skeletal muscle growth [32] in chicks. More studies concluded that a combination of lights could improve the production of chicks [33]. However, this study shows the effect of GL stimuli on promoting both body weight and breast muscle weight during the embryonic period and after hatching [4,33]. This stimulating effect of monochromatic GL on skeletal muscle may be related to the levels of IGF-1 in the plasma and muscle of chicks [32,34]. Halevy [35] demonstrated that the administration of rhIGF-1 affects muscle development in embryos. Circulating IGF-1 in the blood originates primarily from the liver [36]. Therefore, the regulation of muscle growth may be mediated through the effects of light on liver development. However, the liver plays only a minor role in the supply of IGF-1 to the developing embryo, whereas on hatching and during the post-hatch period, it is the main source of IGF-1 for the chicken. GL photostimulation may lead to a high level of IGF-1 because of increased activation of growth hormone (GH) and its liver receptors [4]. This effect, in turn, results in greater activation of the liver to express the *IGF-1* gene. This mechanism of GH connecting to liver growth hormone receptor (GHR) may cause an increase in liver IGF-1 [37]. GL photostimulation of embryos at 0 days old increased plasma GH, hypothalamic growth hormone-releasing hormone (GHRH), liver GHR, and insulin-like growth factor-1 (IGF-1) mRNA levels [38]. Dishon [39] showed that GL promotes arylalkylamine N-acetyltransferase (AANAT) mRNA expression and the secretion of melatonin in the chick pineal gland. Melatonin may play a crucial role as an antioxidant in the liver and other tissues [40,41]. The mechanism through which this improves embryo development could be explained by the fact that melatonin induces the activity of the detoxifying enzyme, glutathione peroxidase, in several chick tissues. As a consequence, hydrogen peroxide is metabolized and thereby reduces the generation of highly toxic hydroxyl radicals [4,41]. As such, melatonin should be considered a component of the antioxidative defense system in avian species. Thus, GL exposure can enhance chick pinealocytes and retinal cells to express AANAT mRNA and to secrete melatonin, which may be dependent on promoting c-Fos expression and cell proliferation [39,42,43]. Birds exhibit photoreceptors at two major sites: The retina (retinal photoreceptors) and in areas of the brain, such as the hypothalamus, pineal, and olfactory bulbs (extra-retinal photoreceptors). Both sites begin developing at 12 days of incubation as shown by mRNA expression and immunohistochemistry. These sites are related to activation of neurotransmitters such as glutamate, serotonin, and dopamine, and they are also associated with activation of the thyroid axis through activation of certain enzymes, such as Type II iodothyronine deiodinase. Liang [44] showed an increase in GHRH expression from 16 days of incubation until 1-day post-hatching. GL photostimulation from ED15 increased hypothalamic GHRH mRNA expression to that of the positive control [5,45].

Accordingly, this may explain the increase in brain weight, neurons, and BI observed in the groups exposed to light compared with the control group. Conversely, the increase in brain weight may result from the development and increased body weight because a direct correlation exists between body weight and brain weight [46]. However, how light can reach the fetus of mammals that live within their mother’s body can be questioned. The possibility that light influences the establishment or the modulation of later bias in mammals is still under scrutiny [47]. Therefore, we recommend conducting further studies on these animals.

## Conclusion

The exposure to different light colors, such as RL, BL, and GL, but especially GL, exhibits a significant effect on embryonic development as observed through changes in embryo weight, body weight, and hatchability, as well as neurophysiological traits such as neurons, brain weight, and BI.

## Authors’ Contributions

SMA: Designed the *in vitro* and *in vivo* experimental design, participated, and supervised the execution of the experiment, and contributed to the writing of the manuscript. MAA and SSS: Conducted the histopathological examinations and contributed to the writing of the manuscript. AAM and MQA: Participated in the execution of the experiment and contributed to the writing of the manuscript. AT: Participated in writing of the manuscript, data collection and statistical analysis. All authors read and approved the final manuscript.
